# Chromatin‐associated condensates as an inspiration for the system architecture of future DNA computers

**DOI:** 10.1111/nyas.15415

**Published:** 2025-09-05

**Authors:** Lennart Hilbert, Aaron Gadzekpo, Simon Lo Vecchio, Mona Wellhäusser, Xenia Tschurikow, Roshan Prizak, Barbara Becker, Sandra Burghart, Ewa Anna Oprzeska‐Zingrebe

**Affiliations:** ^1^ Institute of Biological and Chemical Systems Karlsruhe Institute of Technology Eggenstein‐Leopoldshafen Germany; ^2^ Zoological Institute Karlsruhe Institute of Technology Karlsruhe Germany

**Keywords:** 3D genome organization, architecture of the cell nucleus, biological phase separation, DNA nanotechnology, synthetic biology

## Abstract

The genome stores and processes approximately 1.5 gigabytes of encoded information. In this article, we propose that the eukaryotic genome and its adaptable three‐dimensional packing in the form of chromatin offer a valuable template for the system architecture of DNA‐based digital computers. We examine embryonic and stem cells, which exhibit distinct chromatin‐associated condensates enriched in transcription machinery. These dynamic biomolecular condensates facilitate the spatial association of genes, genomic control elements, and molecular machinery responsible for reading the genomic code. Drawing a compelling analogy to the von Neumann computer architecture—which integrates storage, processing, and memory in most electronic computers—we reflect on how the operational principles of these condensates could inspire the design of a similar architecture for future DNA computers. In particular, we describe how one could recreate such an architecture by exploiting the process of surface condensation, which underlies the formation of chromatin‐associated condensates. We conclude by reviewing our initial steps of constructing synthetic DNA nanostructures that follow the same operational principles and enable programmable surface condensation. Finally, we outline how computational methods from accelerated materials design could further advance the development of DNA computer system architectures.

## BACKGROUND: DIGITAL PROGRAMMING AND DATA STORAGE USING DNA

Deciphering the genetic code has led to an understanding of DNA as the blueprint of life, encoding and controlling the fundamental functions of cells and of our own bodies. At the same time, the possibility of using DNA for digital information processing was recognized. Early successes include the transfer of canonical algorithmic problems into genetic code.^1^ Here, the solution of the famous Traveling Salesman problem proved experimentally that certain classes of problems can be transferred to DNA sequences and solved more efficiently due to the error‐prone, highly parallel replication of DNA.^2^ Extrapolating from these early experiments, it was recognized already in 1994 that

One can imagine the eventual emergence of a general‐purpose computer consisting of nothing more than a single macromolecule conjugated to a […] collection of enzymes that act on it.^1^

In a more contemporary perspective: DNA, as a linear macromolecule, could play the role of a digital information carrier that is acted upon by enzymes that implement the read, write, and logic operations needed to establish a general computer.

**Table:** 

Frequently asked question	Our answer
The genetic code consists of four letters, GTCA, and digital code of the two values 0 and 1. How can this work out?	Two bits can encode the combinations 00, 01, 10, and 11, which can be used to represent the four letters GTCA. Considering that GC and TA are matched by hydrogen bonds, longer stretches of DNA can also be used to encode 0 or 1 as high GC or high AT content.
When will DNA computers be faster than electronic computers?	DNA‐based computers are unlikely to be faster than electronic computers, except for extremely parallel problems. Modern electronic CPUs operate in the GHz range, meaning 1 billion operations per second. For comparison, a complete induction‐transcription‐termination cycle of a gene is estimated to occur in one or even a few minutes.^3^, ^4^, ^5^, ^6^ Accordingly, the speed of sequential (nonparallelized) computation is unlikely to be improved by DNA computers. The more important technological advantage is that DNA can directly interface with cellular and molecular biomedical and biotechnological processes, which electronic computer chips can not.
What will the screen and keyboard of a DNA computer look like?	Most likely, DNA computers will carry out tasks that do not need much direct human interaction, such as controlling cells inside a patient's body during cancer therapy or intelligent control of the bioproduction of chemicals. This is not much different from electronic computers: most electronic computers and chips are embedded in machines or operate in closed‐off server farms. These electronic computers are also almost never directly controlled by a human at a screen.

Although not representing a general computer, several widely visible examples of biomedical technology can be described as programmable. The response against the global COVID‐19 pandemic was, in part, supported by new RNA vaccine technologies, allowing a “digital‐first,” accelerated vaccine production. Modern cancer immunotherapies also include the genetic programming of immune cells to recognize specific amino acid code patterns. Lastly, even the human genetic code has been manipulated in a targeted fashion, tackling disease‐causing code errors^7^ and raising fundamental ethical questions.^8^

**TABLE 1:** Representative examples of recent progress in DNA computing architectures.

Example architectures	Implications for DNA computing
Recording of cancer biomarkers,^9^ developmental signaling time courses,^10^ and digital video data^11^ into DNA memory registers	Recording of time‐resolved signals and complex data for later retrieval by sequencing represents the state of the art, but requires the destruction of the DNA to recover information
DNA‐based implementation of modular Field‐Programmable Gate Arrays (FPGAs)^12^	Example of sourcing a key concept from electronic computers to direct the design of system architecture of DNA‐based computers
DNA‐based implementation of single neurons and prototypical architectures as needed for artificial neural networks^13^, ^14^, ^15^	Illustrates the possibility of building purpose‐specific hardware components using DNA as a computational substrate
Acceleration of logical computation by embedding DNA‐based logical gates in liquid‐like condensates^16^	Proof‐of‐principle that a system architecture that emulates intracellular organization can improve key aspects of computing performance

One area of astonishing progress is the use of DNA for digital information storage. The recognition of the high storage density and long‐term stability of DNA together with new technologies for in vitro synthesis and high‐throughput sequencing of DNA enabled first demonstrations of the feasibility of encoding, editing, and retrieving digital information.^17^, ^18^, ^19^, ^20^ Subsequent work further increased storage density and introduced crucial features necessary also for conventional computer hard drives, such as error‐free information retrieval and full random access capability.^21^, ^22^ These developments reveal a fundamental functional discrepancy between cellular processes that rely on genomic DNA and digital memory as used in electronic computers. In electronic memory, read and write performance is balanced. In biological cells, read and copy operations are well‐implemented via transcription and replication, respectively, whereas template‐free write operations are rarely encountered. This lack of well‐performing “write functionality” is an area of ongoing work. Emerging technical adaptations of CRISPR/Cas9‐based genome editing have pushed DNA‐based storage to the recording of intracellular states^23^ and even digital video data^11^ in the genomes of living bacteria, demonstrating the inherent biocompatibility of DNA information storage. In rapid succession, the recording of extracellular signals into genomes of mammalian cells was also demonstrated.^10^, ^24^ At this point in time, DNA as data storage has entered mainstream technology, with a vast array of techniques covering the key steps of encoding, writing, preservation, retrieval, reading, and decoding.^25^ Of note, the storage of an entire episode of a TV series in DNA has also been accomplished.^26^, ^27^

## STATE OF THE ART: SYSTEM ARCHITECTURE AS AN EMERGENT CHALLENGE IN THE CONSTRUCTION OF A GENERAL‐PURPOSE DNA COMPUTER

Despite the progress in using DNA for information storage, the construction of a general‐purpose computer remains to be achieved. In the development of electronic computers, the definition of a well‐working system architecture was pivotal. In our opinion, defining an effective system architecture might play an equally decisive role for the construction of robust and efficient DNA‐based computers. A pragmatic and effective approach is to provide the overall system architecture by a combination of microfluidic liquid handling and automation via conventional computers.^28^ This approach, however, requires frequent interfacing between electronic and molecular components, instead of remaining ìn‐DNA for the majority of tasks. Remaining with mostly DNA as a substrate for the computation itself, as well as the overall system architecture, is increasingly recognized as a key challenge required for computations within living cells and also within running biotechnological setups.^12^ This challenge has only been recognized for some years, so progress is still in an exciting early phase. Fundamentally different avenues to finding such system architectures are currently being explored.

One avenue toward the construction of a DNA‐based system architecture is the consideration of historical turning points in the development of the architecture of electronic computers. In particular, the transition from logical circuits constructed for a specific purpose to general‐purpose logical circuits that can be programmed for particular tasks must be mentioned. In this direction, a DNA‐based equivalent of a Field‐Programmable Gate Array (FPGA) has been achieved as a major step toward general programmability^12^ (Table 1). FPGAs are computer chips that can be reprogrammed to perform different tasks by changing their internal logic, allowing for customization without requiring external modification of the hardware. In the case of DNA‐based FPGAs, the implementation of Uniform Transmission Signals, which are also essential for electronic FPGAs, served as a crucial design concept. Another major turning point in the development of electronic computer hardware is represented by executing massively parallel computations on specialized hardware with a limited set of allowed operations, that is, Graphics Processing Units (GPU). GPUs, initially developed to accelerate graphics rendering, are now a cornerstone for the training of artificial neural networks. DNA‐based hardware that implements hierarchical layers equivalent to those of artificial neural networks has also been demonstrated, using a combination of DNA‐based logical circuits with microfluidic transfer of dissolved DNA^12^, ^13^, ^15^ (Table 1).

Another fruitful avenue is to first consider the natural limitations of using biomolecules for digital computing. Reliable and fast calculations in conventional computers are based on the transfer of electrons within semiconductors. In stark contrast, DNA computing relies on biomolecules that aimlessly wander in small numbers in the random motion of diffusion, making interactions between such molecules inherently slow and unreliable. These physical constraints are fundamental obstacles to the construction of fully functional DNA computer hardware. However, they are also the same challenges that have been overcome by biological evolution of, for example, cellular signaling and metabolic pathways. Here, a central concept from the structural biology of multiprotein enzyme complexes is the placement of the molecular components in a well‐defined binding geometry. Such an arrangement can dramatically increase reaction efficiency and substrate specificity. Indeed, using precisely folded DNA strands, known as DNA origami, as nanometer‐scale architectural scaffolds for components of DNA logical gates, the computation process was rendered more modular, controllable, and efficient.^29^ Encapsulating DNA strands that carry out logical operations in water‐permeable vesicles is another example in which a life‐like compartmentalization allows increased stability, modularity, and even parallelization of computation.^30^ Embedding the DNA components of logical circuits within liquid‐like droplets that resemble intracellular condensates has also accelerated the rate of computation^16^ (Table 1). These examples represent one emerging approach, which is to use concepts of cellular architecture as an inspiration for the system architecture of future DNA computers (Table 1).

## RATIONALE: CHROMATIN‐ASSOCIATED CONDENSATES AS AN INSPIRATION FOR THE SYSTEM ARCHITECTURE OF A DNA COMPUTER

The intracellular processes that might most closely approximate the requirements of DNA computing are those that support the processing of genomic information. The particular approach we will further outline in this article is to investigate the architecture and operation principles of the readout of genetic code in embryos and stem cells as an inspiration for the system architecture of future DNA computers. The readout of genetic information proceeds via the selective transcription of genetic code sequences in the form of DNA into shorter sequences in the form of RNA. In the human genome, the protein‐coding parts of its approximately 20,000 genes make up about 2% of the overall genomic sequence. The remaining, noncoding fraction contains an estimated 100,000 to 1 million so‐called enhancers.^31^ Enhancers are regulatory elements that control which genes are, in fact, transcribed. In early embryonic development and during stem cell differentiation, the set of transcribed genes undergoes rapid and wide‐ranging changes, orchestrated largely via enhancers as the responsible control elements. These dynamic, global, yet reliable changes in transcription are frequently called a “change of gene expression program.” From the perspective of modern computers, these transitions could be understood as changing between different software programs, or “app switching,” an operation typical of most modern operating systems.

**FIGURE 1:**
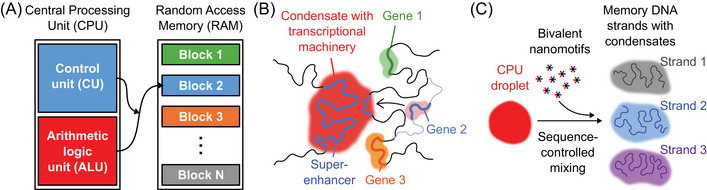
An analogy between the architectures of electronic computers and transcription factories. (A) Von Neumann computer architecture, in which a CPU is connected to different blocks in a working memory. (B) A transcription factory centrally stores the molecular transcription machinery and can selectively access genes. (C) The operation principle of a CPU‐memory architecture based on synthetic DNA nanostructures.

In the remainder of this article, we will explain our perspective on how the three‐dimensional organization and dynamics of the stem cell and early embryonic genome can instruct proof‐of‐principle system architectures for future DNA computers. We discuss the underlying processes of liquid‐like condensation and how these relate to the targeted activation and repression of developmental genes. This discussion reveals an apparent likeness between the enhancer‐based control of developmental genes and the von Neumann architecture, which describes how the Central Processing Unit (CPU) is connected to the memory in practically all modern‐day electronic computers (Figure 1B). Developmental genes undergo controlled visits to clusters of transcriptional machinery, which accumulates at active developmental enhancers (Figure 1A). In the von Neumann architecture, a Control Unit dynamically connects the CPU to one of the addresses in the working memory (Figure 1B). This conceptual likeness is also the starting point for the cell‐free, fully synthetic reimplementation of such a von Neumann architecture. Here, sequence‐programmable condensation of DNA nanostructures on synthetically produced DNA strands forms the basis for an address‐based CPU‐to‐memory routing architecture (Figure 1C). We review first results from this approach to building a von Neumann‐like architecture. We close by outlining how virtual‐experimental hybrid approaches can enable a path toward a first implementation of such an architecture.

To render our approach feasible, we deliberately forego other crucial areas of technical development required for general computing: molecular components for sequence‐based logic and information storage, information encoding paradigms, algorithm development, or overall system instruction management. Our reasoning is that these areas will, with near certainty, continue to progress rapidly without our own direct contribution. We rather hope to contribute to the thinking of how to integrate these components into a well‐thought‐out system architecture under the inevitable constraints on DNA as a material substrate for computation.

## STEM CELL‐SPECIFIC CONDENSATES ESTABLISH A UNIQUE ARCHITECTURE TO CONTROL THE READOUT OF GENETIC INFORMATION

An understanding of the genome sequence as a code of life is widely considered common knowledge. How the genome is stored and continuously rearranged in the three‐dimensional space of the cell nucleus is less widely known, but is the focus of much ongoing genomics research. The concept of transcription factories, proposed in the 1990s (Figure 1A), describes one exciting aspect of this highly dynamic 3D packaging of the genome.^32^, ^33^ These factories differ from a conventional picture in which molecules approach and then bind to target genes to control their transcription. Instead, genes move toward stationary clusters containing these molecules, where transcription is then activated.^34^ Although the concept of transcription factories drew much attention, it was quickly met with criticism because transcription factories could not be extracted from cells and directly identified. In recent years, however, the concept of transcription factories has received renewed attention, largely on the basis of new perspectives on how cells can form highly dynamic, liquid‐like assemblies.^35^ These biomolecular condensates form similarly to the phase separation of water and oil. In the case of transcription factory formation following cell division,^36^ as well as optically controlled intracellular phase transitions,^37^ the separation of specific molecules into highly concentrated condensates occurs within seconds or minutes. The dissolution of such droplets is similarly rapid. Accordingly, detection using biochemical methods that are based on permanent binding between molecular species is difficult.

**FIGURE 2:**
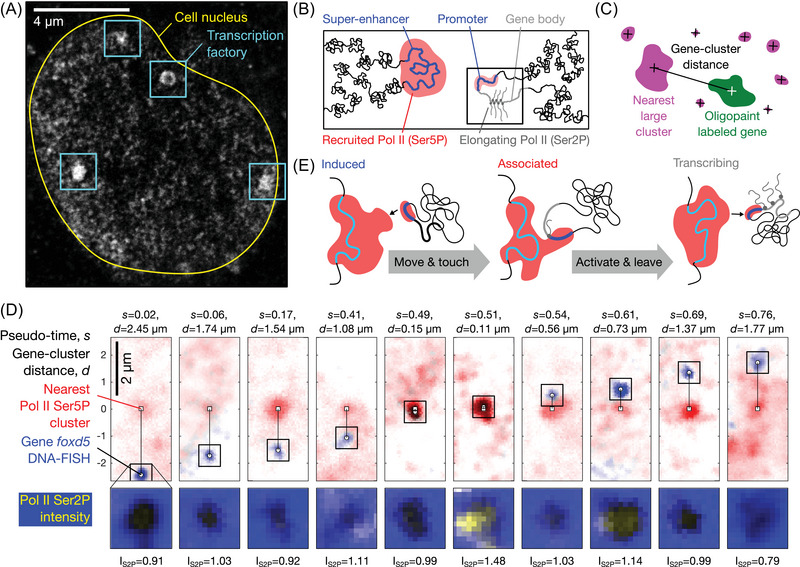
Transcription factories as hubs of gene control. (A) Microscopic image of RNA polymerase II in the nucleus of a zebrafish embryo, obtained by STEDD super‐resolution microscopy, sourced from Ref.^38^. (B) Sketch of the interaction of a super‐enhancer (left) and a gene (right) for the control of transcription via touching of the promoter to the super‐enhancer‐associated condensate. (C) Sketch of image‐based quantification of the distance from a given gene to the nearest polymerase cluster. (D) Algorithm‐based sorting of microscopy images into pseudo‐time series reconstructs the visit of genes to clusters.^6^ (E) Sketch of the main stages of the observed association, activation, and detachment of genes.

Conceptualizing transcription factories as intracellular, liquid‐like condensates has, indeed, significantly renewed our understanding of their formation and function. Many molecular species involved in the control of gene readout tend to form liquid‐like droplets.^39^, ^40^ RNA polymerase II—the enzyme that reads protein‐coding genes in animal and plant cells and transcribes them into RNA—undergoes a process similar to droplet formation before it begins the actual readout process.^3^ A clear tendency toward droplet formation has also been observed for transcription factors that control the activation and deactivation of certain genes.^41^ This tendency has been observed in living cells, particularly stem cells, where RNA polymerase II and transcription factors co‐occur in distinct condensates (Figure 2A).^38^, ^39^ Thus, the original concept of transcription factories is experiencing a renaissance, enabled by the new understanding of droplet‐like assemblies in living cells.

The relevance of this new understanding of transcription factories becomes especially clear when considered in the context of embryonic development and stem cells. Any mistakes in transcription control can lead to significant developmental abnormalities or altered cell fate decisions, rendering precise and rapid gene activation essential. Transcription factories supposedly enable the control over gene expression via the formation of gene loops in three‐dimensional space, bringing together genetic sequences that are far apart on a chromosome (Figure 2B).^42^ These loops often connect DNA sequences that act as control elements, so‐called enhancers, with the target genes they control. In embryos and stem cells, multiple enhancers can associate in space and provide the basis for the assembly of exceptionally long‐lived transcription factories.^43^ These factories maintain high local concentrations of proteins called transcription factors that, together with enhancers, control the activation of genes.^42^ This long‐lived assembly of control elements in a factory can then be visited by different genes (Figure 2D,E).^6^ After genes have undergone activation, they leave the factory in an active state to complete the readout of the DNA sequence outside the factory (Figure 2D,E).^3^, ^4^, ^5^, ^6^, ^39^, ^43^, ^44^ Transcription factories thus organize the targeted activation of genes by concentrating various control elements in one place. Access to the DNA sequence information of different target genes is then achieved by pulling them into the factory. In different cell types, for example, in developing embryos, different genes can be pulled into these factories and their activation can be efficiently and precisely controlled.^45^, ^46^

**TABLE 2:** Functionalities afforded by transcriptional condensates, which could benefit the operation of DNA‐based computers.

Functionality afforded by transcriptional condensates	Expected possibility for DNA‐based computing
Dynamic sequestration of limited transcription machinery on a limited set of target genes and genomic control regions^47^, ^48^	Dynamic context generation by controllable condensation of read/write and logic machinery on specific DNA memory segments
Establishment of reliable control from selective but short‐lived binding of transcription factors via local concentration increase^49^, ^50^	Conversion of stochastic, single‐molecule interactions into quasi‐deterministic outcomes required for reliable computation
Selective partitioning of transcriptional regulators based on charge‐patterned regions^51^	Dynamic allocation of read/write machinery to memory blocks defined by different DNA address sequences

The organization of transcription control in factories visited by genes offers several functional advantages, which should also be beneficial with respect to the challenges of DNA computation. Sequestration within transcription factories can concentrate crucial transcription machinery in a location‐dependent manner. In the example of the pluripotency factor Nanog and the transcription control enzyme CDK9, this local concentration was found to have a dual benefit. On the one hand, the concentration of Nanog and CDK9 in condensates termed “transcription bodies” results in the effective activation of embryonic transcription.^47^ On the other hand, the sequestration of transcription factors such as Nanog within these transcriptional condensates prevents the aberrant activation of off‐target genes.^48^, ^49^ In addition, the high concentration within transcriptional condensates can transform short‐lived binding of transcription factors into a quasi‐permanent occupation, as the unbinding of one molecule is almost instantaneously followed by the rebinding of another molecule of the same type.^49^ In effect, stochastic binding events are converted into quasi‐deterministic activation. The reliable activation within factories as well as the prevention of leaky off‐target activation correspond well with the requirements of deterministic computation and selective processing of specific parts of DNA‐encoded data (Table 2).

The selective partitioning of transcription factors into specific condensates has recently been attributed to charge patterning in the intrinsically disordered regions of these proteins. Intrinsically disordered regions are flexible protein segments that lack a fixed three‐dimensional structure. This flexibility enables dynamic interactions and is frequently required for phase separation.^51^ The charge patterning of amino acids clearly describes how the intrinsically disordered regions of a protein can be programmed to encode the partitioning into specific types of condensates. In the context of a computer, targeting read and write functionality to specific regions of memory in a dynamic, programmable fashion is a crucial prerequisite for the execution of algorithms and programs (Table 2). The dynamic connection of read/write machinery to specific addresses of memory also points toward a more general similarity between the operation principles of transcription factories and already existing electronic computers, which we will discuss in the following section.

## ANALOGY TO THE VON NEUMANN ARCHITECTURE FOR DYNAMICALLY CONNECTING A PROCESSOR TO DIFFERENT MEMORY ADDRESSES

The von Neumann architecture was a decisive conceptual breakthrough in the historical development of the electronic computer. In its essence, this architecture is still the foundation for how central processors connect to the working memory in the absolute majority of current electronic computers. The CPU of a computer provides highly optimized read, write, and logic hardware at a single location in an electronic computer. This hardware can then be connected on demand and at high speed to any desired address in the working memory (RAM) (Figure 1A). This architecture allows memory with a large capacity to be used flexibly by a single CPU and ensures stringently controlled operation of the computer with a clearly defined state.

The transcription factories described in the previous section have many properties that are necessary for the interaction of a CPU with an address‐based working memory. For example, all crucial functions for controlling the activation of genes are centrally located in the transcription factories (Figure 2B). Furthermore, certain genes can be specifically transported to these factories, leading to their selective readout (Figure 1B). Considering that transcription factories offer advantages due to their condensate nature (Table 2) and are functionally analogous to the canonical von Neumann computer architecture, they provide a promising avenue toward designing a well‐working architecture for a DNA‐based computer. A logical further development of our research into the organization and functioning of cellular transcription factories is, therefore, the construction of artificial DNA systems that implement the same architectural and operational principles.

Note that the analogy with a von Neumann architecture applies specifically to embryonic and stem cells, which harbor long‐lived transcription factories.^39^, ^43^, ^45^, ^52^ In differentiated cells, short‐lived clusters of transcription factors form and dissolve within several seconds, prior to transcriptional activation.^3^, ^5^, ^53^, ^54^ This on‐demand clustering contrasts with a permanently available CPU and will thus not be further discussed in this article. Such a high level of adaptability might, however, benefit other technical systems. Here, a loose analogy can be drawn with on‐demand spawning of processes or threads, a common feature of modern operating systems. Further, embryonic and stem cells exhibit not only one, but multiple, long‐term stable transcription factories.^39^, ^52^ The occurrence of multiple factories could be likened to modern computer or GPU architectures comprising multiple processors that work in parallel, pointing toward avenues for future work. Lastly, the stochasticity of gene‐cluster visits remains a key discrepancy. For general computation, the state of the computation must be unambiguous and the choice of the memory address must be deterministic and temporally coordinated with the computation. These challenges seem to have been overcome in the process of olfactory receptor choice, where exactly one out of 2000 receptor genes becomes transcriptionally active by interaction with a super‐enhancer cluster, while all other receptor genes become transcriptionally repressed.^55^

## SURFACE CONDENSATION AS AN OPERATION PRINCIPLE FOR TRANSCRIPTION CONTROL

**FIGURE 3:**
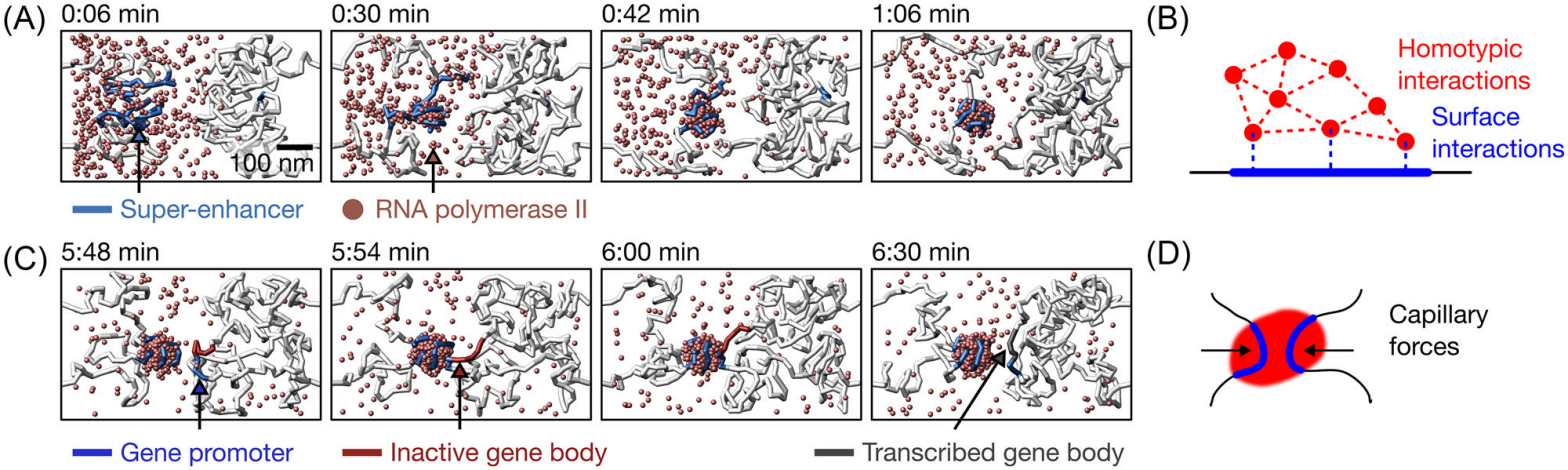
Surface condensation as a process that underlies the formation of transcription factories and subsequent gene‐factory visits. (A) Formation of the transcription factory by surface condensation on a regulatory genomic region. RNA polymerase II should be understood to also represent other molecular transcription machinery. (B) A combination of homotypic and surface‐directed interactions is required for surface condensation. (C) The gene forms a liquid bridge with the transcription factory and breaks off from the factory after being activated. (D) Capillary forces can compact and zip together chromatin strands.

Although the analogy between the biologically observed transcription factories and the CPU in the von Neumann architecture is conceptually informative, it does not yet provide concrete approaches for establishing such an architecture. Based on recent work, the targeted condensation of molecular factors at specific genomic regions, called surface condensation, offers crucial insights into the process of transcription factory formation.^38^, ^39^ The role of surface condensation can be intuitively understood from computer simulations of the interaction of a model gene with one transcription factory. In these simulations, a transcription factory is formed by the condensation of particles that represent transcription machinery on a super‐enhancer region (Figure 3A). This region has a locally constrained affinity for the transcription machinery, leading to an increased concentration in the vicinity of the super‐enhancer. The increased local concentration facilitates condensation, leading to the formation of a clearly visible cluster. The formation of this cluster is soon followed by pulling in of the super‐enhancer genomic region. After sufficient time of simulation has passed, one can see the cluster of particles that contains the super‐enhancer region (Figure 3A), matching experimental observations in stem cells and embryos.^38^, ^39^

From this simulated formation of a factory, a few crucial aspects of the process of surface condensation can already be concluded. While surface condensation leads to the formation of a liquid‐like condensate, it differs from the more widely known process of liquid–liquid phase separation (LLPS) in a crucial aspect. In canonical LLPS, only the interactions between a single species of particles (homotypic interactions) are considered, and the condensation into liquid‐like droplets occurs when the bulk (spatially averaged) concentration exceeds the saturation concentration. For surface condensation to occur, particles are present only at subsaturated bulk concentrations, and only locally exceed the saturation concentration by being attracted by a surface. The particles must, therefore, exhibit not only homotypic interactions, but also surface interactions (Figure 3B). These interaction rules were confirmed for stretched DNA in in vitro experiments with a single transcription factor, which is targeted to specific regions of the stretched DNA strand by recognition of target sequence motifs.^41^ The same general scenario was also found in the formation of transcription factories in pluripotent cells of developing embryos, where epigenetic marks of regulatory regions, represented by H3K27ac, take on the role of a condensation surface.^38^ These observations relate to a more general observation of non‐sequence‐specific, but enhancer‐versus‐promoter specific accumulation of proteins on chromatin,^56^, ^57^ potentially involving G‐quadruplex formation.^58^ Here, it already becomes evident that surface condensation can constrain condensation to specific target sites, while spurious condensate formation throughout the bulk volume is prevented. This aspect of surface condensation can increase the fidelity of transcriptional control and has been found to apply especially in developing embryos (Table 3).^48^, ^49^

As one continues with the simulation past the formation of a transcription factory, toward the visit by a gene, additional contributions of surface condensation to the control of transcription become apparent. As the gene promoter explores the simulation space, it occasionally comes close the transcriptional cluster (Figure 3C). By forming a “liquid bridge,” the gene promoter is then pulled toward the cluster. Based on our theoretical model, this pulling together of two strands can be categorized as an effect of capillary action: cohesion within the polymerase II‐enriched liquid condensate is rooted in homotypic interactions, whereas adhesion to the enhancer and promoter region is rooted in surface interactions (Figure 3B,D). Capillary forces have recently been discussed in terms of their general ability to organize the genome in three dimensions.^59^ Also, more specifically, the ability of such liquid condensates to zip together distal strands of chromatinized DNA was demonstrated in reconstituted assays in egg extracts.^60^ It is especially this sticking together of different genomic regions that is required to establish selective connections between transcription factories and the genes that are to undergo activation by visiting a transcription factory.

**FIGURE 4:**
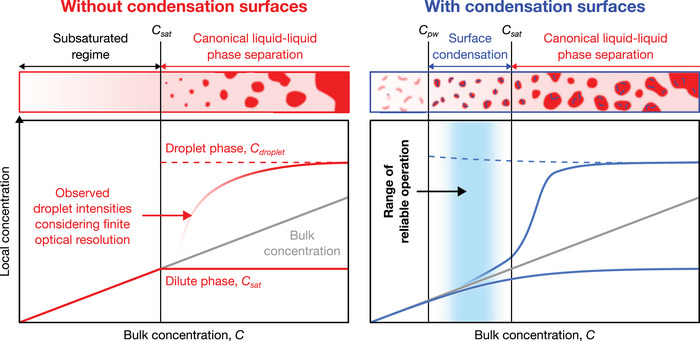
Delineation of the surface condensation regime in contrast to canonical liquid–liquid phase separation (LLPS). Reliable operation of a computing system that uses properties and functions afforded by surface condensation can be expected within the inner regions of the surface condensation regime, above the pre‐wetting concentration ($C_{pw}$) and below the saturation concentration ($C_{sat}$). Blue and red lines in the bottom diagram represent stable concentrations that can be obtained by demixing into condensates via surface condensation or LLPS, respectively. Dashed lines are theoretical expectations and solid lines are expected observations considering a finite microscope resolution. Sketch based on simulations in our previous work.^38^

A more systematic distinction between surface condensation and canonical LLPS also reveals the specific conditions under which proper system operation can be expected.^41^ Both surface condensation and LLPS lead to demixing, referring to the establishment of membrane‐less compartments with locally increased concentrations. One of the main control parameters for such demixing is the bulk concentration (C), referring to the concentration of a given molecular species when averaging over the entire space of a given system (Figure 4). As the bulk concentration exceeds the pre‐wetting concentration ($C_{pw}$), the formation of condensates in the presence of condensation surfaces can be observed, even though no formation of droplets occurs away from these surfaces. Only as the bulk concentration further increases above the saturation concentration ($C_{sat}$), the system undergoes canonical LLPS: a decomposition into droplets with an elevated internal concentration ($C_{droplet}$) and a dilute phase with a concentration of $C_{sat}$ occurs. Thus, while surface condensation offers unique properties that differ from canonical LLPS (Table 3), these can only be reliably used as a basis for system operation within the concentration range that leads to surface condensation, $C_{pw}<C<C_{sat}$ (Figure 4).

**TABLE 3:** Differences between surface condensation and canonical liquid–liquid phase separation.

Surface condensation	Liquid–liquid phase separation
Condensates can form at concentrations below those required for canonical liquid–liquid phase separation	Concentration for liquid–liquid phase separation must be exceeded
Formation of condensates at specific target sites	Formation of droplets throughout entire system volume
Condensate volume limited primarily by affinity and number of target sites	Condensate volume limited by global availability of droplet material
Number of condensates controlled by number of surfaces	Droplets coarsen and fuse over time, while small droplets vanish

## FAST‐TRACKING HARDWARE DEVELOPMENT WITH SEQUENCE‐PROGRAMMABLE, SYNTHETIC DNA NANOSTRUCTURES

**FIGURE 5:**
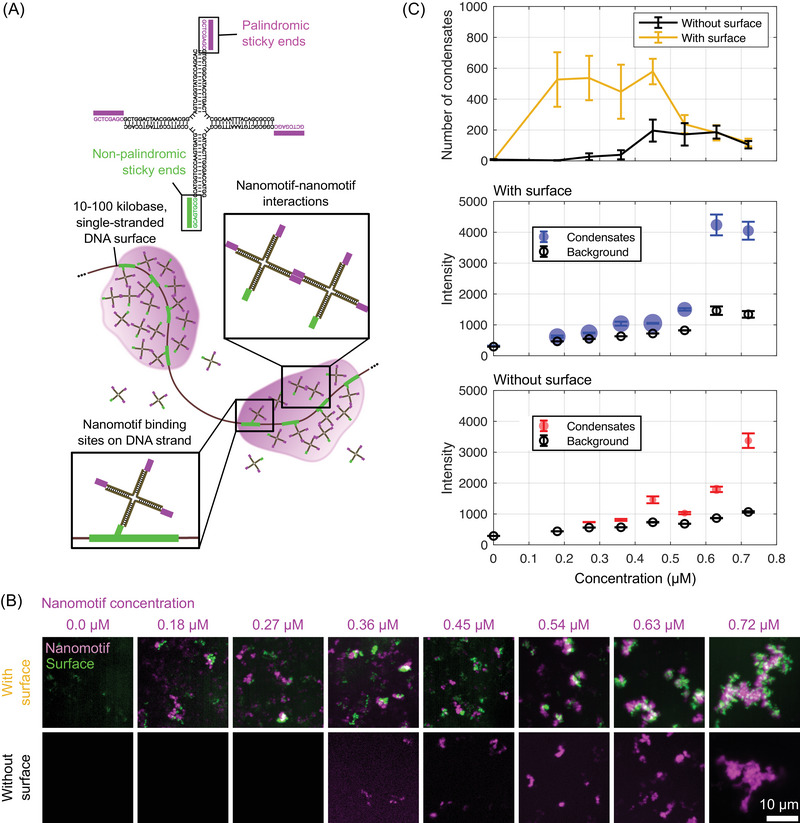
Use of synthetic DNA nanostructures to reconstruct the surface condensation process underlying transcription factory formation. (A) The condensed material of a transcription factory is mimicked using X‐shaped nanomotifs made of short DNA oligomers. The nanomotifs have sequence‐encoded binding sites for other nanomotifs (magenta) and for target areas located on DNA strands (green). These target areas enable the local formation of surface condensates, similar to the condensate formation at genomic control elements. (B) Two‐color microscopy images showing nanomotifs (magenta), to which surfaces (green) were added or not added. Images are maximum intensity z‐projections, intensity look‐up tables were adjusted for each image individually to facilitate visual interpretation. For separate channel images, see Figure S1. (C) Number of condensates per volumetric stack, the nanomotif channel intensity within these condensates, and the nanomotif channel background intensity for increasing nanomotif concentration, in the presence or absence of added condensation surfaces. All values are mean with 95% confidence intervals, 500 bootstrap resamples, n=20 volumetric stacks per condition for condensate number and background intensity; for condensate intensity, n is the total number of condensates pooled from 20 stacks per condition. For the middle and bottom intensity plots, the circle area is proportional to the number of detected condensates.

Ultimately, DNA‐based computer hardware should be integrated into living cells to reach its full potential. By dispensing with the cellular context, however, cell‐free systems based on commercially available DNA building blocks^61^ enable the testing of concrete architectures within several weeks instead of several years. An increasingly widely used foundation for such architectures is provided by DNA nanostructures that form phase‐separated droplets and can be selectively fused or separated in a DNA sequence‐programmable fashion (Figure 5A).^61^, ^62^ These nanostructures are created by annealing single strands of DNA into star‐shaped, multivalent particles that form networks via complementary DNA overhangs (Figure 5A). The properties of such condensates can be tuned by changing external parameters, such as temperature or salt concentration, by modifying the DNA sequences, or by fusing proteins to the DNA nanostructures.^61^ By incorporating reaction processes, for instance, in the form of strand displacement, the condensates' internal organization can be dynamically controlled.^63^, ^64^ Molecules tagged with DNA sequence “address labels” can be targeted into specific droplet species.^65^ Localization of reactants and the controlled fusion of droplets provides control over reactions and increases reaction rates.^66^ The detection of tumor marker miRNAs can be transduced into demixing of droplets via logical gates,^67^ demonstrating that DNA nanostructures are indeed a suitable platform for bioinspired computation.^68^

Even though phase‐separating DNA nanomotifs can, in principle, be used to rapidly validate and develop many types of architectures, we focus our work on architectures mimicking key operation principles of transcription factories. As a first proof of principle, we have used phase‐separating DNA nanostructures to mimic the subtle relationship between transcriptional activity of genes and the shape of transcription factories these genes interact with: factories, in which genes are activated and subsequently ejected at a high frequency, appear distinctly more fragmented than those with little such activity.^69^ We constructed amphiphile nanomotifs to mimic the previously proposed amphiphilic effect of transcriptionally active genes. Using high‐resolution fluorescence microscopy, automated image analysis, and simulations of DNA nanomotifs, we showed that transcription factories in pluripotent zebrafish embryos and artificial DNA droplets undergo the same basic fragmentation process with increasing amphiphile concentrations.^69^ This proof‐of‐principle study establishes that, indeed, targeted rebuilding of architectures and operation principles observed in the cell nuclei of embryos is possible.

Another crucial feature of transcription factories is the selective condensation on specific areas of the genome. Also, in zebrafish embryos, we were able to show that transcription factories are formed by targeted condensation at genomic control elements (super‐enhancers) (Figure 2A).^38^ These genomic regions bear molecular markers that support surface condensation. We have now been able to implement the same principle of surface condensation using artificially generated DNA strands (Figure 5A). Here, it is possible to induce surface condensation of DNA nanomotifs on DNA strands with matching target sequences (Figure 5A). To ensure that the system constructed in this fashion, in fact, does exhibit surface condensation, we assessed condensate formation by confocal microscopy over a range of nanomotif concentrations, in the presence and absence of condensation surfaces. A visual inspection of example images as well as a comprehensive quantification, indeed, reveal the expected concentration regime in which condensates form in the presence of surfaces, but do not form in the absence of surfaces (Figure 5B,C). Equally, as expected, for higher nanomotif concentrations, the appearance and number of nanomotif condensates in the presence and absence of surfaces become similar (Figure 5B,C). The interpretation that, at higher nanomotif concentrations, condensate formation becomes independent of surfaces is further supported by the occurrence of condensates that appear largely devoid of surfaces (Figure 5B). The onset of condensate formation should theoretically result in a jump to a much higher and near constant nanomotif concentration inside the condensates (Figure 4). Experimentally, however, a progressive increase of condensate intensity is visible (Figure 5C). We attribute this discrepancy to effects of finite microscope resolution and will confirm this explanation below, using simulations. In combination, our observations and quantification show how the addition of condensation surfaces can shift the occurrence of condensates from the canonical saturation concentration, $C_{sat}$, to a lower concentration, $C_{pw}$, thereby establishing the theoretically expected surface condensation regime. Constructing a purely DNA‐based system that can be driven into the desired surface condensation regime represents a first, small, but clear step toward building an architecture for interfacing a DNA‐based processor and working memory.

## ACCELERATED DESIGN OF ARCHITECTURES BY EXPERIMENTAL‐COMPUTATIONAL HYBRID APPROACHES

**FIGURE 6:**
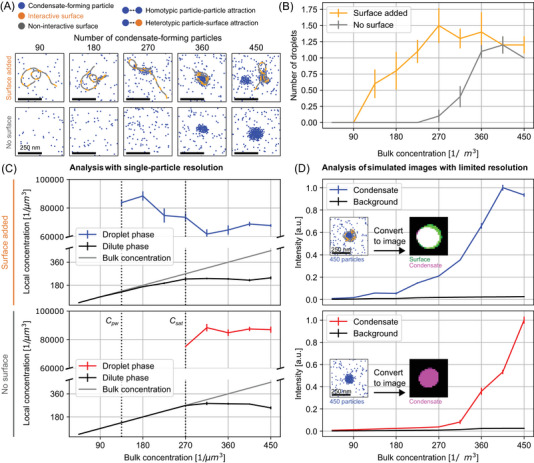
Simulation of surface condensation in the presence of a model polymer chain. (A) Basic interaction rules in a system of diffusing particles result in liquid–liquid phase separation and surface condensation. 2D‐projections of simulations show that for a low number of condensate‐forming particles, droplets only form on interactive polymer sections. Only at higher numbers of condensate‐forming particles can droplets form independently. (B) The association of particles into droplets is detected at lower particle concentrations in the presence of a condensation surface. (C) When increasing the bulk concentration of condensate‐forming particles above the pre‐wetting concentration, $C_{pw}$, a dense droplet phase appears in the presence of a condensation surface (top). Without a surface, a dense droplet phase forms only above the higher saturation concentration $C_{sat}$. Droplet phase concentration obtained using the convex hull around DBSCAN detected clusters. (D) Simulating the limited resolution of a microscope and implementing a quantification of condensate intensities similar to the analysis of microscopy images leads to a progressive increase of intensity above $C_{pw}$ (in the presence of surface, top) or above $C_{sat}$ (without surface, bottom). All values are mean ± SEM, n=10 independent simulations per condition.

The design of computing systems, as well as high‐tech devices in general, is frequently supported by computational methods. In the field of accelerated materials design, close feedback of computational approaches with design and evaluation has become a key asset.^70^ Similarly, synthetic DNA nanostructures can be represented computationally, bridging scales from all‐atomistic, via base‐pair resolution, to coarse‐grained micrometer‐scale simulations.^71^, ^72^ This computational representation can be illustrated for the surface condensation scenario. Here, self‐attracting particles and polymer surfaces with alternating interactive and noninteractive block segments serve as a coarse‐grained model of the X‐shaped motifs and DNA surface strands, respectively (Figure 6A). In these simulations, the polymeric surfaces can, indeed, support the formation of particle clusters at concentrations where these particles alone do not form clusters (Figure 6B). Simulations of this kind can also support a more comprehensive evaluation, revealing specifically the concentration range in which surface condensation occurs and reliable system operation can be expected. When a surface is present, particle concentrations split into a dilute phase and a dense phase at the concentration $C_{pw}$ (Figure 6C, top). In the absence of a surface, the split into a dilute and a dense phase occurs only at a higher concentration ($C_{sat}>C_{pw}$, Figure 6C, bottom). The simulations, in line with our theoretical expectations (Figure 4), illustrate how the surface condensation range is delimited by $C_{pw}$ and $C_{sat}$. When we introduce effects of the finite resolution of a microscope, our simulation results also correspond well with our experimental results (Figure 5C). In particular, condensates predominantly occur above the concentration $C_{pw}$ (with surface) or $C_{sat}$ (without surface), and progressively increase in intensity with increasing particle concentration (Figure 6D). The correspondence with experimental observations can be seen also in another specific behavior: above $C_{sat}$, surfaces localize to a subset of condensates, so that some of the condensates appear largely free of surface (Figures 5B and 6A). Taken together, our simulations consolidate our theoretical understanding of how the interplay between homotypic and surface interactions can lead to surface condensation.

The wider applicability of such coarse‐grained simulations can be seen in another example, where we clarified the working mechanism of amphiphilic nanomotifs, ultimately allowing us to mimic the dispersal of transcriptional condensates in embryonic cells.^69^ To increase the realism of coarse‐grained simulations, the model parameters can be inferred by scale‐bridging approaches. For example, individual DNA nanomotifs can be simulated at base‐pair resolution using the oxDNA package.^73^, ^74^ Iterative fitting methods, such as Bayesian optimization, can efficiently request the most highly informative coarse‐grained models, speeding up parameter inference by orders of magnitude.^75^, ^76^ Ultimately, one can imagine that the actual production and experimental testing of DNA nanostructures will also be included into such a closed optimization loop, offering the full promise of accelerated design of DNA‐based computing architectures. Such an optimization loop could start with several designs for components of the DNA‐based system architecture. For example, multiple X‐motifs with different sticky‐end sequences that should form distinct condensates on surfaces, thus serving as system memory. Rather than exploring the full design space through experiments alone, one could screen for promising designs with computational methods. Once the subset of candidate designs has been evaluated experimentally, simulation parameters can be corrected, a revised set designs can be obtained, and again fed into the computational screening step. This loop, alternating between experimental validation and increasingly well‐adjusted simulation parameters, should significantly improve the efficiency of finding a suitable sequence‐level design.

## FUTURE CHALLENGES AND OPPORTUNITIES

In closing, we look ahead, naming some challenges that come more clearly into focus after we have proposed a DNA‐based von Neumann architecture. As for electronic computers, the number of logical circuits and memory blocks that can be integrated into a single chip limits algorithmic complexity and also sheer performance. In electronic computers, this limit is mainly imposed by how finely circuitry can be laid out in terms of feature size, now commonly on the single nanometer scale. In DNA‐based computing hardware, first examples of scaling the number of integrated circuits has demonstrated that the limit currently is imposed primarily by the establishment of modular sets of sequences that do not interfere with sequences in other modules and well‐defined message passing between such modules.^12^ Also, in electronic computers, increasing algorithmic complexity relies on error correction codes that are built into all underlying operations. In DNA computers, working on the basis of biochemical reactions, the error rate is an even more acute challenge and has been addressed, for example, via the Redundant Residue Number System^77^ or DNA nanostructures that implement the Hamming code for error correction.^78^ The operation of a conventional CPU is based on the repetition of fetch‐decode‐execute cycles. Reliably sequential cycles for a DNA‐based CPU could potentially be established exploiting operation principles underlying recent observations of controlled formation and subsequent dissolution of transcriptional microgels.^79^ Long‐term storage in data centers is often achieved by more persistent storage media, such as magnetic tape. For DNA nanomotifs, a reversible encapsulation of specific DNA nanomotifs in a long‐term stable core‐shell configuration and recall by toehold‐mediated displacement was demonstrated, offering a potential approach for similar functionality.^80^

Unique opportunities are offered by the inherent evolvability of biological systems. Both the artificial evolution of DNA sequences and saturation mutagenesis in cell populations can provide massively parallel optimization, similar to, for example, the application of genetic algorithms in the training of artificial neural networks. The localized hypermutation of antibody‐coding regions in adaptive immunity could provide inspiration for how to rapidly generate string‐type variables that can be screened in immunotherapy applications. Each of these challenges illustrates the potential for technology development offered by the operating principles observed in the biological cell nucleus, and simulation models and virtual design approaches provide a bridge from these conceptual starting points toward the construction of actual DNA‐based computing systems.

## MATERIALS AND METHODS

### Microscopy‐based analysis of transcription clusters and gene‐cluster visits

The microscopy image in Figure 2A is adapted from Ref. 38. The microscopy data in Figure 2D were prepared using an improved version of the image analysis code already documented in our previous publication.^6^ The code is developed on GitHub https://github.com/lhilbert/VisitorGene_PseudoTime and the version used to generate the figure shown here is available as a snapshot release: https://doi.org/10.5281/zenodo.14870263. The underlying image data as well as the complete methods for sample preparation and image acquisition are also documented in our previous publication^6^ and are publicly available: https://doi.org/10.5281/zenodo.5266995.

### Simulation of gene‐cluster visits

The simulations in Figure 3 were produced using custom‐developed simulation code on the basis of LAMMPS and Python, available via GitHub https://github.com/lhilbert/EnhancerShoeBox. Simulation parameters: chromatin monomer diameter σ=60nm; 400 Pol II Ser5P particles; length of both chromatin segments = 400 monomers; promoter length = 3 chromatin monomers; gene length = 5 monomers; shoe box length = 12 monomers (720nm); activation rate = $30\;\min^{-1}$; Ser5P threshold for activation = 80.

### Titration experiments with DNA nanomotifs

**Oligonucleotide design**. Nanomotif sticky end and backbone sequences for a four‐ended nanomotif were sourced from previous work.^61^ The 8‐nt sticky end sequence of one of the four sticky ends was adjusted to be nonpalindromic, allowing for hybridization with target sequences on long DNA strands produced by rolling circle amplification (RCA), resulting in a redesigned “Nanomotif X” (NX in our laboratory's nomenclature). During the design of the nonpalindromic end, the melting temperature was kept consistent. For RCA, we considered previously suggested template strand lengths from 13 to 105 nt^81^ and chose to design a template of 68‐nt length. Four consecutive repeats of the binding sequence of the fourth, nonpalindromic nanomotif were positioned in the center of this template (“Surface X4,” SX4 in our nomenclature). To bridge the open ends of the linear template into a circle, a 20‐nt bridging element was designed that is complementary to the first and last 10 nucleotides of the template sequence. Note that this bridge element, after circularization, also serves as a primer for ϕ29 polymerase. Besides the binding target sequences, the template allows the binding of 20‐nt “blocker strands,” which render their target sequence inaccessible for unintended hybridization. The blocker strands are conjugated with a fluorescent dye, additionally allowing their localization in fluorescence microscopy. All strands were designed using the Life Science R&D cloud Benchling and purchased from http://biomers.net. The different strand sequences are listed in Table 4. The dried single DNA oligomer strands were suspended in 1× Tris‐EDTA buffer (TE) to a concentration of 200 µM and kept in a –20°C freezer for long‐term storage.

**TABLE 4:** Strand names and sequences for nanomotifs, surface structures, and primers.

Strand name	Sequence
X‐1_8	GCTCGAGCGCTGGACTAACGGAACGGTTAGTCAGGTATGCC AGCAC
X‐2_8	GCTCGAGCGTGCTGGCATACCTGACTTTCGCAAATTTACAG CGCCG
X‐3_8	GCTCGAGCCGGCGCTGTAAATTTGCGTTCATCACTTGGGAC CATGG
X‐4_8	GCAGTGCG CCATGGTCCCAAGTGATGTTCCGTTCCGTTAG TCCAGC
X‐2_Atto647N	Atto647N‐GTGCTGGCATACCTGACTTTCGCAAATTTACAG CGCCG
Surface X_4rep	P‐TGAGTGTATGACTATAACGCAGTGCGGCAGTGCGGCAG TGCGGCAGTGCGTAACAAATACTGTAGTAC
Bridge X	CATACACTCAGTACTACAGT
Blocker X	Atto 488‐ACTGTAGTACTGAGTGTATG

*Note*: P indicates phosphorylation.

**Nanomotif preparation**. The single DNA oligomer strands were prepared by diluting the DNA oligomer stocks (200 µM) 40‐fold in nanomotif buffer (2× TE and 700 mM NaCl) to a concentration of 5 µM in a PCR tube (Table 5). For one strand, 10% were substituted with a DNA oligomer for which the 8‐nt sticky end was replaced by the fluorescence dye Atto‐647N, to enable the detection of nanomotif accumulations by fluorescence microscopy (Table 5). Using an Eppendorf Mastercycler X50, the tubes were first held at 85°C for 3 min, followed by decreasing the temperature at a rate of –1°C/min down to 40°C, and holding at 40°C until using the prepared tubes for further preparation of mixtures for the titration experiment.

**TABLE 5:** Strand names and concentration of oligonucleotides.

Strand name	Concentration
X‐1_8	5.00 µM
X‐2_8	4.50 µM
X‐3_8	5.00 µM
X‐4_8	5.00 µM
X‐2_Atto647N	0.50 µM

**RCA production of condensation surfaces**. To obtain circularized templates for RCA, the template and the bridge DNA oligomer were prediluted in 1× TE buffer to a working concentration of 25 µM. A ligation reaction mix was prepared, comprising 2 µL T4 ligase buffer, 0.8 µL linear template DNA oligomer (20 pmol), 1.6 µL bridge DNA oligomer (40 pmol), 1 µL T4 DNA ligase (400 U, stock concentration 400,000 U/mL), and 14.6 µL $H_{2}O$. Enzymes and buffer solutions were purchased from New England BioLabs (NEB). The solution was gently mixed, briefly spun in a pipetting centrifuge, and subsequently incubated at room temperature (RT) for 10 min. Enzymes were then heat‐inactivated at 65°C for 10 min. Circularized templates were purified after cooling to RT using the DNA Clean & Concentrator‐25 kit (DCC‐25, Zymo Research).

For the actual amplification, a reaction mix was prepared comprising 30 µL of the liquid containing the purified circularized DNA, 5 µL of 10× Φ 29 buffer, 1 µL of Φ 29 DNA polymerase (10 U, from stock concentration 10,000 U/mL), 0.8 µL of dNTPs (10 mM stock concentration), and 13.2 µL $H_{2}O$. Buffer, enzyme, and dNTPs were purchased from NEB. The reaction mix was incubated for 1.5 h at 30°C. Enzymes were then heat‐inactivated at 65°C for 10 min. After allowing the reaction mix to cool down to RT, the samples were washed three times by briefly spinning in a pipetting centrifuge, aspirating 35 µL of supernatant, and adding back in 35 µL of $H_{2}O$, resulting again in a final volume of 50 µL.

**Final preparation of samples**. For each titration concentration, PCR tubes with a total volume of 13.75μl were prepared (for an overview, see Table 6). To this end, the volume of prepared X‐nanomotif was gradually substituted by nanomotif control buffer (10 µL 1× TE, 40 µL $H_{2}O$, 50 µL of 2× TE/700 mM NaCl) to create a concentration titration series. The $H_{2}O$‐based eluted liquid containing RCA‐generated surfaces was always added in a volume of 8.5μl. For without‐surface control conditions, 8.5μl of $H_{2}O$ were added instead. For all conditions, 0.25μl of an Atto‐488‐labeled fluorescent blocker DNA oligomer (200 µM stock concentration in 1× TE buffer, 3.64 µM final concentration in the mixture) were added. The samples corresponding to the different conditions were prepared for microscopy recording in a randomized order, all experiments were repeated on two different days. One concentration condition (0.11 µM) was excluded from further analysis due to a technical failure in one of the repeats resulting in aberrantly high numbers of condensation surfaces for only that concentration (Table 6). All tubes were heated up to 60°C for 2 min to allow rearrangement of the surfaces and the nanomotifs while the nanomotifs are in the liquid phase,^61^ followed by subsequently decreasing the temperature at a rate of –1°C/min to 40°C, and keeping samples at this temperature until imaging.

**TABLE 6:** Volumes of different ingredient‐containing solutions used to prepare the different conditions of the titration experiment.

Surface	$H_{2}O$ control	Nanomotif	Buffer control	Blocker	[Nanomotif]	Repeats
8.5	0	2.0	3.0	0.25	0.72 µM	2
8.5	0	1.75	3.25	0.25	0.64 µM	2
8.5	0	1.5	3.5	0.25	0.55 µM	2
8.5	0	1.25	3.75	0.25	0.45 µM	2
8.5	0	1.0	4.0	0.25	0.36 µM	2
8.5	0	0.75	4.25	0.25	0.27 µM	2
8.5	0	0.5	4.5	0.25	0.18 µM	2
8.5	0	0.25	4.75	0.25	0.091 µM 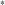	1
8.5	0	0.0	5.0	0.25	0.0 µM	2
0	8.5	2.0	3.0	0.25	0.72 µM	2
0	8.5	1.75	3.35	0.25	0.64 µM	2
0	8.5	1.5	3.5	0.25	0.55 µM	2
0	8.5	1.25	3.75	0.25	0.45 µM	2
0	8.5	1.0	4.0	0.25	0.36 µM	2
0	8.5	0.75	4.25	0.25	0.27 µM	2
0	8.5	0.5	4.5	0.25	0.18 µM	2
0	8.5	0.25	4.75	0.25	0.091 µM 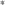	2
0	8.5	0	5.0	0.25	0.0 µM	2

*Note*: Volumes are stated in µL and add up to a total volume of 13.75 µL in all conditions. 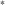 Condition excluded from quantitative analysis because only a single technically valid repeat was available for the with‐surface condition for this nanomotif concentration.

**Preparation of microscopy slides**. Glass microscope slides and cover slips (#1.5, selected thickness) were coated by covering them with 2% BSA solution. After 5 min of incubation time, the remaining solution was wicked away with a chemical wipe, followed by air drying. Cover slips and slides were then stuck to each other using double‐sided adhesive tape to form flow channels. After injecting a given channel with a sample, the channel was sealed with silicone grease.

**Microscopy**. The samples were visualized at 21°C using a VisiTech iSIM microscopy with a 40× water immersion objective (Apo LWD 40× WI λ S DIC N2, NA 1.15). Images were recorded using dual ORCA‐Flash4.0 V3 cameras with simultaneous two‐channel acquisition to avoid displacement of objects due to consecutive acquisition of color channels. The image data underlying Figures 5 and S1 are part of a publicly available data set: https://doi.org/10.5281/zenodo.15231478.

**Image processing**. Fluorescence microscopy images were processed using a combination of intensity‐based thresholding and clustering methods. To distinguish relevant structures (condensates and surfaces) from the background, we applied a threshold based on the mean (μ) and standard deviation (σ) of pixel intensities for each fluorescence channel. Objects were detected as connected components formed from pixels with intensity at least four times σ above μ. The remaining pixels were assigned to the background. After thresholding, connected components are identified using an 8‐connected neighborhood criterion. Objects with volumes less than 0.05 µm^3^ were removed to reduce the influence of image noise. To account for spatial clustering of condensates, we applied Density‐Based Spatial Clustering of Applications with Noise (DBSCAN) with a cut‐off distance of 4 µm and retention of all cluster sizes.

Local intensity for both condensates and background were extracted using a custom algorithm that defines random regions of interest (ROIs) on 2D z‐projection images. For each experimental condition, ROIs (100×100 pixels) were randomly sampled within a central 700×700 pixel region of the image. Individual condensates within each ROI were identified using connected component analysis, and the mean pixel intensity was computed separately for condensates and background regions.

The image processing code is available publicly on GitHub: https://github.com/lhilbert/SurfacesAndCondensates/releases/tag/v0.1.0

### Simulation of condensation of DNA nanomotifs

The simulations in Figure 6 use ReaDDy for Brownian dynamics of interactive particles. Condensate‐forming particles interact with each other and binding sites on surfaces via a harmonic well potential with a minimum at 20nm. Other particle interactions are repulsive, modeled with a harmonic potential for distances below 20nm. Particles thus have a radius of roughly 10nm. Surfaces are polymers comprising 120 monomers. Sections of four attractive monomers are separated by sections of eight repulsive monomers. Bond and angle potentials are soft harmonic potentials between groups of two and three monomers, respectively. To speed up condensate formation, particles were initially placed in a spherical volume with a radius of 200nm. Harmonic potentials keep all particles inside the simulations box with edge lengths of 1μm. Total simulation time was $5\cdot 10^{6}$ steps of 0.5ns, at a temperature of 40°C. Droplets are detected as clusters of condensate‐forming particles by DBSCAN, using a minimum sample number of 10 and maximum distance of 40nm. To calculate the dense phase concentration (or intensity) inside the droplets, two different methods were used. The first method calculates the volume of the convex hull around DBSCAN‐detected clusters, expanded at the nodes by the particle radius. The second method simulates the limited resolution of a microscope. First, the simulation box is discretized into voxels of 20nm edge length. Next, the number of particles per voxel is used as the voxel intensity, which is subsequently blurred with a Gaussian filter with standard deviation of 3 voxels. Condensates and image background are then separated by applying Otsu's thresholding method^82^ to the intensity histogram. For all methods, n=10 repeats were used to obtain mean values and the standard error of the mean. The implementation is based on code used in Ref. 69. Jupyter notebooks to run and evaluate the simulations are available on GitHub: https://github.com/aaron‐gad/Condensate_BD_Sim

## AUTHOR CONTRIBUTIONS

LH: Conceptualization, writing—original draft preparation, supervision. LH, MW, and AG: Writing—review and editing. LH, AG, MW, and SLV: Formal analysis. AG, XT, BB, and SLV: Investigation. LH, AG, RP, EAO‐Z, SB, and SLV: Software. LH, AG, XT, RP, EAO‐Z, BB, and SB: Visualization.

## COMPETING INTERESTS

The authors declare no conflicts of interest.

## PEER REVIEW

The peer review history for this article is available at https://publons.com/publon/10.1111/nyas.15415.

## Supporting information



Figure S1: Fluorescence images of DNA nanomotifs and DNA surfaces.
