# Supplementary material for: Chromatin‐associated condensates as an inspiration for the system architecture of future DNA computers
Source: Ann N Y Acad Sci. 2025 Sep 5;1552(1):12–28. doi: 10.1111/nyas.15415 (PMC12576880; doi:10.1111/nyas.15415)
Supplement: Supplementary file 1 — Figure S1: Fluorescence images of DNA nanomotifs and DNA surfaces. [file NYAS-1552-12-s001.pdf]

Supplemental Material

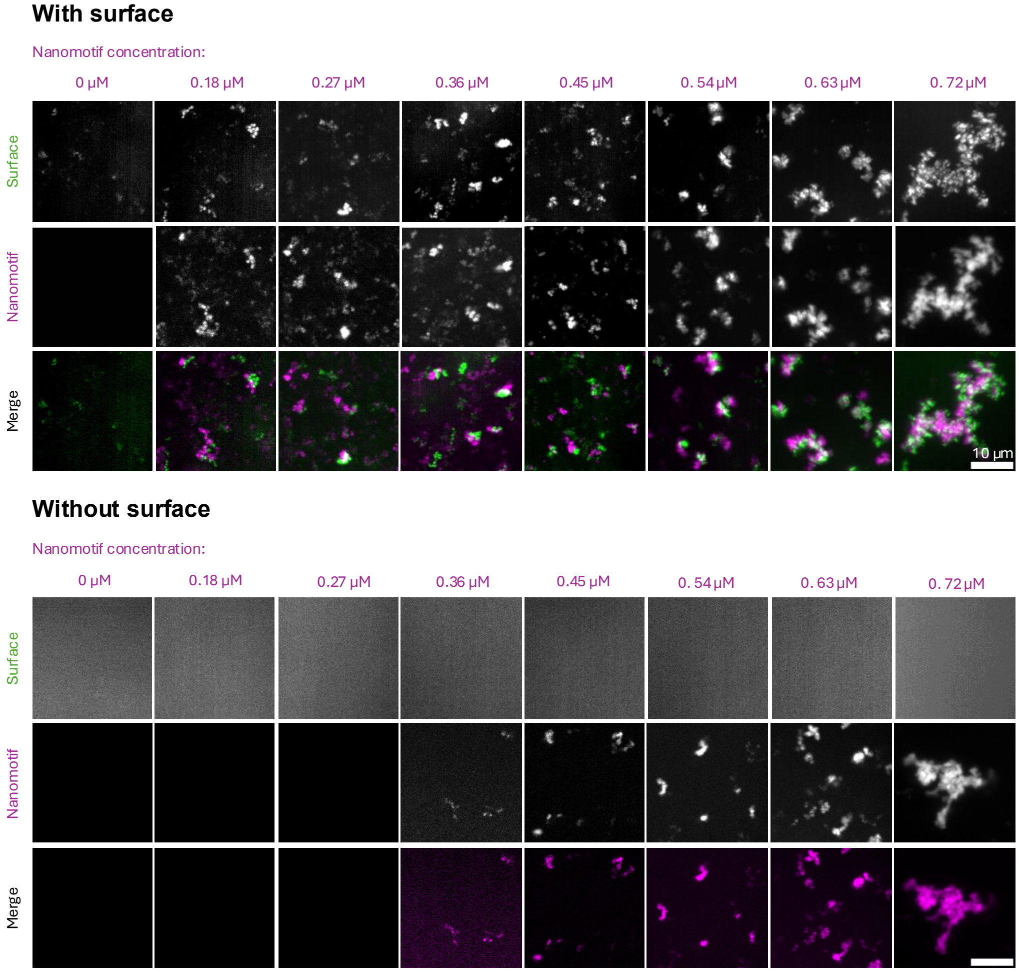

Supplemental Figure S1: **Fluorescence images of DNA nanomotifs and DNA surfaces.** DNA surfaces (green) promote the condensation of DNA nanomotifs (magenta) below the saturation concentration. In contrast, phase separation occurs only at the saturation concentration without surface. The scale bar represents 10 μm and applies to all images. Images are maximum intensity z-projections, intensity look-up tables were adjusted for each individual image to facilitate inspection of depicted objects.
